# Supplementary material for: Altered microRNA Transcriptome in Cultured Human Liver Cells upon Infection with Ebola Virus
Source: Int J Mol Sci. 2021 Apr 6;22(7):3792. doi: 10.3390/ijms22073792 (PMC8038836; doi:10.3390/ijms22073792)
Supplement: Supplementary file 1 [file ijms-22-03792-s001.zip › Supplementary_File/C_ GO_Analysis_Results/16-30nt_go_Makona-96h-Huh7_vs_Control-96h-Huh7_down.mature_mirna_targets/MF_result(Human).html]

| GO.ID | Term | Ontology | Count | Pop.Hits | List.Total | Pop.Total | Fold.Enrichment | Pvalue | FDR | Enrichment.Score | Gene.Ratio | GENES |
| --- | --- | --- | --- | --- | --- | --- | --- | --- | --- | --- | --- | --- |
| GO:0005515 | protein binding | Molecular function | 387 | 11565 | 518 | 17548 | 1.13360876162433 | 7.06720517072515e-06 | 0.00915909790125979 | 5.15075230033373 | 0.747104247104247 | CPLX1//LRRK2//CPLX3//GOSR2//ELK1//TEAD1//BHLHE40//PASD1//GNAO1//GNAZ//PROK1//NUCB1//THBS1//DPP4//KIT//SLC6A3//RNF125//MAEA//LMOD1//DBNL//NF2//PARVA//EPS8L2//SPTBN2//WAS//MLPH//MYO19//SHANK3//DIXDC1//MICAL2//TIRAP//PARD6B//ARHGEF4//RALGPS2//RALGDS//TIAM1//ARHGEF6//IQSEC3//DLG4//FGFR3//FGFR2//GRIN2B//NCAM1//BCR//TAC4//TNFSF4//TNFSF14//NDP//SFRP1//WNT11//EDN1//BMP10//IL1B//ITGA5//RASL11B//GPNMB//EMP2//TLN1//JAM3//FERMT3//SEMA7A//FSHB//CCK//UNC13B//CAMKK2//CTH//EWSR1//NRGN//PPP3CB//KCNQ5//KCNQ4//HTRA4//CXCL14//PTPRU//PTPRT//CTNND2//CALCOCO1//SKP1//DYNC1I1//KIF2A//CCDC88C//DAPK3//MAPRE1//RBFOX1//PRKAA1//SH3GL1//YWHAB//FGF11//ALDOC//PACSIN1//KRT2//MED6//TRIB1//HEYL//NFATC4//NFKB1//INSIG2//PIM1//MED1//PURA//PAXBP1//CRTC1//RAF1//LARP1//MAPKAP1//CDC42EP1//SNPH//CCDC6//SH3BP5//FZD2//TRABD2B//FZD3//SMAD1//RALBP1//EXOC4//UBE4B//COPS5//SIRT3//HMOX1//KMT2E//PEX5//TNKS2//TSPAN14//TTC28//PARP16//CDKN1B//CDKN2A//SLC12A7//PAK2//RHOF//PRKAB1//MAPK4//USP37//TRIM5//CARHSP1//PPP1R26//PPP1R3F//ARFIP2//TGM2//SRSF9//SYT2//SYT9//SYT14//ABCC8//CD74//DBI//SHISA9//SEMA4G//NRIP1//DNAJB12//CUX1//TOMM34//RNF217//RNF114//SLC25A5//PML//RTN4//UBE2Z//HLTF//PALM//REEP2//SEPT7//UBXN2A//VLDLR//FOXP3//TRIM68//SPIN3//PIK3IP1//SEMA5B//ASF1B//RAG1//DAZL//DLAT//ALAD//P2RX2//ESYT1//SULT4A1//MCIDAS//AQP1//SH3GLB2//CREBZF//SNRPA//TRIM21//CNTN2//BEST1//DDX39B//PIP4K2C//RNF135//DDI2//OXSR1//MYOM3//EXD1//IMPA2//CCDC103//ASCL1//MAP3K9//PRTFDC1//TTK//CREB3L3//NFS1//CD79A//HIC1//BEX4//ARL4C//UBXN2B//TP53INP2//PFDN1//KCNIP3//KCNIP2//SCN3B//MMP24//EIF4G2//CC2D1A//LRRC59//UBFD1//SKIL//EFNA3//TAF8//RALGAPB//ANO4//ANO3//GLRX//TAB1//ARHGDIB//BRK1//NDFIP1//S100A12//ACTR3//ACTR2//AKAP10//PTAFR//DNAJB5//NKAIN1//DPYS//TOMM40L//DLX5//PRDM4//PRDM12//RGMA//CD81//TIMM23//PYURF//EMC8//PPIH//AGPAT1//TGOLN2//ARID3B//EBP//FERMT2//RASSF1//VPS45//CHRNA3//AP1S1//SLAIN1//UBXN10//LYPD6//TPRA1//GLYCTK//SYNPR//PACRGL//LSM11//NEK7//CSN3//USP54//TET3//FBXO45//CYB561A3//VASH1//FOXG1//KLHDC10//ZBTB43//FAM168A//IQCE//MGRN1//SATB2//ATP1B4//HAAO//ZNF281//LDOC1//LCLAT1//NAT9//FBXL3//FBXO10//MYCBP//PTPN18//GPR3//ANKRD52//LIN9//C16ORF72//WDR91//GRM2//CYP2S1//ST8SIA5//TIMM22//NRBF2//CD209//EHD3//CDR2L//HMGB3//HOXC9//FAM92B//IL4R//FAM111B//C16ORF87//LGALS3BP//SNX19//MAGEA11//MEA1//MRC1//ASNA1//SMCO3//MYBL2//NFYA//PAX5//PLLP//PHF20L1//RLIM//UCHL5//BIN2//C9ORF78//POLR2E//DCAF16//PPP1R2//ENOX1//VPS53//DRAM1//CHDH//THUMPD1//MAP7D1//MBNL3//FAM214A//PRSS8//GRIPAP1//PTBP1//TBC1D24//WDFY1//RANBP10//WDR19//PCTP//RBM3//REL//RFX4//CEP85//USP46//SMPD1//SNRPB//TCF19//TMPRSS2//C1QA//U2AF1//UBE2D3//UBE2E1//VSNL1//NPHS2//PAX8//CACNG1//SLMAP//MIS12//CENPO//SLC52A2//TNFAIP8L2//RPAP3//KIAA0319L//NUP214//FOSL1//URM1//TRIM7//CCDC70//KREMEN1//CASP7//FAM160A2//PRADC1//OGT//KLHL22//CGNL1//FBXL20//PARG//CYP4F2//KRT38//SESTD1//SHKBP1//NREP//CD22//SCAMP1//VPS26A//CREB5//RBM8A// |
| GO:0048365 | Rac GTPase binding | Molecular function | 9 | 67 | 518 | 17548 | 4.55056762519449 | 0.000148074259702763 | 0.0839622875307866 | 3.82952042993678 | 0.0173745173745174 | ARHGEF4//EPS8L2//TIAM1//RALBP1//ARFIP2//ARHGDIB//PAK2//BRK1//WAS// |
| GO:0017048 | Rho GTPase binding | Molecular function | 15 | 173 | 518 | 17548 | 2.93726426674404 | 0.000194357147062006 | 0.0839622875307866 | 3.71139948450264 | 0.028957528957529 | ARHGEF4//BCR//EPS8L2//TIAM1//ARHGEF6//CDC42EP1//RALBP1//ARFIP2//ARHGDIB//PAK2//BRK1//WAS//LRRK2//DAPK3//PARD6B// |
| GO:0050839 | cell adhesion molecule binding | Molecular function | 29 | 489 | 518 | 17548 | 2.00903269614926 | 0.000310469881739562 | 0.085966777335637 | 3.50798052376031 | 0.055984555984556 | GPNMB//EMP2//IL1B//ITGA5//THBS1//TLN1//JAM3//FERMT3//SEMA7A//MMP24//PTPRT//EIF4G2//MAPRE1//LARP1//ARFIP2//DBNL//PAK2//CC2D1A//LRRC59//PARVA//UBFD1//SH3GLB2//RTN4//SH3GL1//EPS8L2//SPTBN2//YWHAB//SEPT7//CDC42EP1// |
| GO:0004842 | ubiquitin-protein transferase activity | Molecular function | 27 | 446 | 518 | 17548 | 2.05081635126478 | 0.000360484781202108 | 0.085966777335637 | 3.44311306542295 | 0.0521235521235521 | UBE4B//RNF38//RNF217//MGRN1//RNF149//RLIM//MED1//RNF125//RNF114//RAG1//SKP1//UBE2Z//HLTF//UBE2D3//UBE2E1//MAEA//FBXO45//FBXL3//FBXO10//TRIM68//KLHL14//RNF123//TRIM21//RNF135//KLHL22//FBXL20//TRIM5// |
| GO:0022839 | ion gated channel activity | Molecular function | 22 | 335 | 518 | 17548 | 2.22472195009508 | 0.000420706429384065 | 0.085966777335637 | 3.37602085089081 | 0.0424710424710425 | P2RX2//GRIN2B//AQP1//ANO4//ANO3//BEST1//CHRNA3//HTR3E//CHRFAM7A//KCNJ12//KCNIP3//SCN3B//SCN1A//SCN7A//CACNG1//CACNA2D2//KCNA6//KCNQ5//KCNQ4//KCNIP2//ABCC8//DLG4// |
| GO:0022836 | gated channel activity | Molecular function | 22 | 338 | 518 | 17548 | 2.20497589728359 | 0.000474766854809649 | 0.085966777335637 | 3.32351960832683 | 0.0424710424710425 | P2RX2//GRIN2B//AQP1//ANO4//ANO3//BEST1//CHRNA3//HTR3E//CHRFAM7A//KCNJ12//KCNIP3//SCN3B//SCN1A//SCN7A//CACNG1//CACNA2D2//KCNA6//KCNQ5//KCNQ4//KCNIP2//ABCC8//DLG4// |
| GO:0005543 | phospholipid binding | Molecular function | 25 | 410 | 518 | 17548 | 2.06563706563707 | 0.000530659119355784 | 0.085966777335637 | 3.27518436803682 | 0.0482625482625483 | SYT9//MARK1//THBS1//TLN1//SYT2//SYT14//WDFY1//TIRAP//MAPKAP1//SESTD1//FERMT2//NRGN//OGT//PCTP//SNX19//TULP4//ARFIP2//UNC13B//ESYT1//PACSIN1//PAFAH2//BIN2//PTAFR//SPTBN2//PLA2G4C// |
| GO:0000982 | transcription factor activity, RNA polymerase II proximal promoter sequence-specific DNA binding | Molecular function | 25 | 415 | 518 | 17548 | 2.04074987207517 | 0.000632617834910757 | 0.0866958987864331 | 3.19885856851555 | 0.0482625482625483 | ARID3B//PRDM4//DLX5//ELK1//SATB2//HEYL//RAX//SMAD1//MYBL2//PAX5//RFX4//HLTF//PAX8//FOSL1//ONECUT2//FOXS1//ZNF281//KCNIP3//ASCL1//NFATC4//CC2D1A//SKIL//BHLHE40//PAXBP1//TEAD1// |
| GO:0045296 | cadherin binding | Molecular function | 21 | 324 | 518 | 17548 | 2.1956956956957 | 0.00066894983631507 | 0.0866958987864331 | 3.17460644819388 | 0.0405405405405405 | CDC42EP1//MMP24//PTPRT//EIF4G2//MAPRE1//LARP1//ARFIP2//DBNL//PAK2//CC2D1A//LRRC59//PARVA//UBFD1//SH3GLB2//RTN4//SH3GL1//EPS8L2//SPTBN2//TLN1//YWHAB//SEPT7// |
| GO:0019787 | ubiquitin-like protein transferase activity | Molecular function | 27 | 472 | 518 | 17548 | 1.93784765394935 | 0.000855335689405157 | 0.100774095769917 | 3.06786340638974 | 0.0521235521235521 | MAEA//RNF38//RNF217//FBXO45//MGRN1//FBXL3//FBXO10//RLIM//TRIM68//RNF114//KLHL14//RAG1//RNF123//SKP1//TRIM21//UBE2D3//UBE2E1//RNF135//KLHL22//FBXL20//TRIM5//UBE4B//RNF149//MED1//RNF125//UBE2Z//HLTF// |
| GO:0000987 | proximal promoter sequence-specific DNA binding | Molecular function | 26 | 451 | 518 | 17548 | 1.95296595296595 | 0.000947678560796482 | 0.10234928456602 | 3.0233389442492 | 0.0501930501930502 | ARID3B//PRDM4//DLX5//ELK1//FOXS1//SATB2//ZNF281//RAX//KCNIP3//SMAD1//ASCL1//MYBL2//NFATC4//PAX5//MED1//CC2D1A//RFX4//SKIL//HLTF//PAX8//FOSL1//PAXBP1//ONECUT2//CREB3L3//BHLHE40//TEAD1// |
| GO:0000978 | RNA polymerase II proximal promoter sequence-specific DNA binding | Molecular function | 25 | 436 | 518 | 17548 | 1.94245687364953 | 0.00127082040908393 | 0.126691019244059 | 2.89591581911924 | 0.0482625482625483 | CREB3L3//ASCL1//BHLHE40//ARID3B//PRDM4//DLX5//ELK1//FOXS1//SATB2//ZNF281//RAX//KCNIP3//SMAD1//MYBL2//NFATC4//PAX5//MED1//CC2D1A//RFX4//SKIL//HLTF//PAX8//FOSL1//PAXBP1//ONECUT2// |
| GO:0005488 | binding | Molecular function | 466 | 15006 | 518 | 17548 | 1.0520075110239 | 0.00146723127761017 | 0.135823695413056 | 2.83350142353016 | 0.8996138996139 | ACBD6//CPLX1//LRRK2//CPLX3//GOSR2//SLC27A4//PRTFDC1//PEX5//MARK1//PGM5//OXSR1//LARP1//HMGB3//CELF4//PURA//NFKB1//CREB3L3//ARID3B//CUX1//PHOX2A//ASCL1//REL//PRDM4//DLX5//ELK1//FOXS1//SATB2//ZNF281//RAX//KCNIP3//SMAD1//MYBL2//NFATC4//PAX5//MED1//CC2D1A//RFX4//SKIL//HLTF//PAX8//FOSL1//PAXBP1//ONECUT2//HEYL//NFYA//TEAD1//NRIP1//FOXP3//CALCOCO1//BHLHE40//PASD1//PTAFR//CD74//GNAO1//GNAZ//PROK1//SYT9//THBS1//TLN1//NUCB1//DPP4//KIT//SLC6A3//RNF125//DPYS//KRBOX1//EHD3//SPRN//ZNF853//ENOX1//ZNF804A//ZNF618//TET3//FOXG1//ZBTB43//CARHSP1//LIN9//PML//POLR2E//CREBZF//RAG1//PRDM12//GPBP1L1//TRIM21//TCF20//CREB5//PRKAA1//CDKN2A//RPP14//DAZL//EXD1//EIF4G2//EWSR1//MAPRE1//SLC25A5//NOVA1//UCHL5//RBFOX1//LRRC59//THUMPD1//MBNL3//UBFD1//RTN4//PTBP1//RBM3//SNRPA//SNRPB//U2AF1//DDX39B//MEX3B//SRSF9//RBM8A//COPS5//MAEA//LMOD1//DBNL//NF2//PARVA//EPS8L2//SPTBN2//WAS//MLPH//MYO19//SHANK3//DIXDC1//MICAL2//TIRAP//PARD6B//ARHGEF4//RALGPS2//RALGDS//TIAM1//ARHGEF6//IQSEC3//DLG4//FGFR3//FGFR2//GRIN2B//NCAM1//BCR//TAC4//TNFSF4//TNFSF14//NDP//SFRP1//WNT11//EDN1//BMP10//IL1B//ITGA5//RASL11B//GPNMB//EMP2//JAM3//FERMT3//SEMA7A//FSHB//CCK//FAXDC2//CYP2C18//CYP2S1//CYP4F3//CYP4F2//S100A12//UNC13B//CAMKK2//SYT2//CAPSL//DGKG//CABP7//SYT14//GALNT3//GPD2//KCNIP2//PPP3CB//MAN1C1//VLDLR//VSNL1//EDEM3//CALN1//CABS1//MED6//TIMM23//PTPRU//ACTR3//PYURF//ARL4C//TRIB1//CDKN1B//EMC8//TAB1//PPIH//AGPAT1//TGOLN2//EBP//RALBP1//TOMM34//FERMT2//PTPRT//CDC42EP1//RASSF1//AKAP10//VPS45//CHRNA3//PIK3IP1//AP1S1//SLAIN1//UBXN10//TAF8//LYPD6//TPRA1//GLYCTK//SYNPR//PACRGL//LSM11//UBXN2B//NEK7//CSN3//CTH//CTNND2//USP54//DAPK3//UBXN2A//SPIN3//DLAT//DYNC1I1//EFNA3//FBXO45//CYB561A3//VASH1//ALDOC//KLHDC10//FAM168A//IQCE//MGRN1//ESYT1//CRTC1//SIRT3//ATP1B4//HAAO//LDOC1//ARFIP2//FZD2//LCLAT1//DNAJB5//SULT4A1//NAT9//FBXL3//FBXO10//MYCBP//PTPN18//GPR3//ANKRD52//C16ORF72//WDR91//GRM2//ST8SIA5//TIMM22//NRBF2//PACSIN1//CD209//CDR2L//HIC1//HMOX1//HOXC9//FAM92B//MCIDAS//IL4R//AQP1//IMPA2//FAM111B//KIF2A//KRT2//C16ORF87//CCDC103//TRABD2B//LGALS3BP//ARHGDIB//SNX19//MAGEA11//MEA1//MAP3K9//MRC1//ASNA1//SMCO3//PAK2//PALM//PLLP//PHF20L1//RLIM//INSIG2//BIN2//C9ORF78//PIM1//DCAF16//PARP16//PPP1R2//VPS53//DRAM1//CHDH//PRKAB1//MAP7D1//ASF1B//BRK1//KMT2E//MAPK4//FAM214A//KCNQ5//PRSS8//GRIPAP1//SH3GLB2//RGMA//TBC1D24//WDFY1//RANBP10//USP37//SEMA4G//WDR19//TP53INP2//PCTP//RAF1//EXOC4//SH3GL1//CEP85//USP46//SKP1//UBE2Z//SMPD1//TCF19//TGM2//TMPRSS2//C1QA//TTK//UBE2D3//UBE2E1//YWHAB//NPHS2//CACNG1//SLMAP//MIS12//MAPKAP1//CENPO//SLC52A2//TNFAIP8L2//RPAP3//FZD3//KIAA0319L//NUP214//CCDC6//TNKS2//NDFIP1//URM1//TRIM7//CCDC70//KREMEN1//CASP7//FAM160A2//PRADC1//RNF135//DDI2//OGT//KLHL22//CGNL1//FBXL20//PARG//TRIM5//KRT38//NFS1//KCNQ4//SESTD1//SHKBP1//NREP//CD22//SH3BP5//SCAMP1//CXCL14//VPS26A//CD79A//CD81//PPP1R26//SEPT7//NRGN//HTRA4//ACTR2//SGK2//UBE4B//TTL//P2RX2//ABCC8//UCK2//PIP4K2C//UCK1//AATK//GBP4//RABL3//RHOF//RERGL//PAFAH2//PLA2G4C//CCDC88C//FGF11//RBMS3//PABPC3//CHRM1//PGGT1B//MMP24//ALAD//TRIM67//TRIM68//ZMIZ1//ZSWIM6//CA7//USP45//DBI//DDAH1//ALDH1A2//SNPH//EVPLL//TSPAN14//TTC28//SLC12A7//PPP1R3F//SHISA9//NPTX2//CLEC1A//GALNT10//CNTN2//DNAJB12//TOMM40L//RNF217//RNF114//REEP2//TULP4//ZNF322//SEMA5B//CHRFAM7A//BEST1//MYOM3//BEX4//LETM2//OTP//TLX2//HOXC6//FOXI2//HIVEP3//PFDN1//SCN3B//RNF38//PRUNE2//NPTXR//STEAP2//RNF149//IDI1//NPTX1//LIMS2//RNF123//TGM4//SNN//BSN//CACNA2D2//KDELR3//RALGAPB//ANO4//ANO3//GLRX//NKAIN1// |
| GO:0070300 | phosphatidic acid binding | Molecular function | 4 | 18 | 518 | 17548 | 7.52809952809953 | 0.0016517980081591 | 0.137356112811083 | 2.78204306192204 | 0.00772200772200772 | MARK1//NRGN//MAPKAP1//SESTD1// |
| GO:0019905 | syntaxin binding | Molecular function | 9 | 93 | 518 | 17548 | 3.27836592352721 | 0.00169575447914917 | 0.137356112811083 | 2.77063702712026 | 0.0173745173745174 | UNC13B//CPLX1//LRRK2//SNPH//SYT2//SYT9//SYT14//CPLX3//ABCC8// |
| GO:0005048 | signal sequence binding | Molecular function | 6 | 47 | 518 | 17548 | 4.3246529203976 | 0.00247115551532647 | 0.188389267521359 | 2.6070999226178 | 0.0115830115830116 | PEX5//NUP214//TIMM22//TOMM40L//KDELR3//KCNIP2// |
| GO:0017075 | syntaxin-1 binding | Molecular function | 4 | 22 | 518 | 17548 | 6.15935415935416 | 0.0035965814471555 | 0.227116534924176 | 2.44411010034312 | 0.00772200772200772 | UNC13B//CPLX1//LRRK2//SNPH// |
| GO:0005216 | ion channel activity | Molecular function | 23 | 424 | 518 | 17548 | 1.83763750273184 | 0.00390254609725962 | 0.227116534924176 | 2.40865195830597 | 0.0444015444015444 | P2RX2//GRIN2B//AQP1//ANO4//ANO3//BEST1//CHRNA3//HTR3E//CHRFAM7A//KCNJ12//CACNG1//CACNA2D2//SCN3B//SCN1A//SCN7A//KCNA6//KCNQ5//KCNQ4//KCNIP2//SLC26A1//KCNIP3//ABCC8//DLG4// |
| GO:0022857 | transmembrane transporter activity | Molecular function | 47 | 1068 | 518 | 17548 | 1.49081746272757 | 0.00403637709615098 | 0.227116534924176 | 2.39400826683374 | 0.0907335907335907 | SLC25A2//P2RX2//GRIN2B//AQP1//ANO4//ANO3//BEST1//CHRNA3//HTR3E//CHRFAM7A//KCNJ12//KCNIP3//SCN3B//SCN1A//SCN7A//CACNG1//CACNA2D2//KCNA6//KCNQ5//KCNQ4//KCNIP2//SLC26A1//ABCC8//SLC6A3//SLC5A3//SLC39A2//SLC30A6//SLC39A8//ATP1B4//SLC35A2//SLC25A5//TIMM22//TOMM40L//SLC5A6//ATP6V1G2//ASNA1//CTNS//SLC23A2//EBP//RALBP1//SLC27A4//DLG4//SLC10A7//SLC12A7//SLC9A5//TIMM23//SLC52A2// |
| GO:0044212 | transcription regulatory region DNA binding | Molecular function | 40 | 877 | 518 | 17548 | 1.54510594647425 | 0.00430031950214596 | 0.227116534924176 | 2.36649927631247 | 0.0772200772200772 | NFKB1//CREB3L3//ARID3B//CUX1//PHOX2A//ASCL1//PURA//REL//PRDM4//DLX5//ELK1//FOXS1//SATB2//ZNF281//RAX//KCNIP3//SMAD1//MYBL2//NFATC4//PAX5//MED1//CC2D1A//RFX4//SKIL//HLTF//PAX8//FOSL1//PAXBP1//ONECUT2//HEYL//NFYA//TEAD1//NRIP1//FOXP3//CALCOCO1//ZNF322//BHLHE40//HIVEP3//TCF20//WNT11// |
| GO:0001067 | regulatory region nucleic acid binding | Molecular function | 40 | 878 | 518 | 17548 | 1.54334614471289 | 0.00438357421533704 | 0.227116534924176 | 2.35817163630059 | 0.0772200772200772 | NFKB1//CREB3L3//ARID3B//CUX1//PHOX2A//ASCL1//PURA//REL//PRDM4//DLX5//ELK1//FOXS1//SATB2//ZNF281//RAX//KCNIP3//SMAD1//MYBL2//NFATC4//PAX5//MED1//CC2D1A//RFX4//SKIL//HLTF//PAX8//FOSL1//PAXBP1//ONECUT2//HEYL//NFYA//TEAD1//NRIP1//FOXP3//CALCOCO1//ZNF322//HIVEP3//TCF20//WNT11//BHLHE40// |
| GO:0000977 | RNA polymerase II regulatory region sequence-specific DNA binding | Molecular function | 32 | 662 | 518 | 17548 | 1.63753222363494 | 0.00444563706182019 | 0.227116534924176 | 2.35206599563673 | 0.0617760617760618 | ARID3B//PRDM4//DLX5//ELK1//FOXS1//SATB2//ZNF281//RAX//KCNIP3//SMAD1//ASCL1//MYBL2//NFATC4//PAX5//MED1//CC2D1A//RFX4//SKIL//HLTF//PAX8//FOSL1//PAXBP1//ONECUT2//HEYL//NFKB1//NFYA//REL//CREB3L3//BHLHE40//CUX1//PHOX2A//PURA// |
| GO:0005057 | signal transducer activity, downstream of receptor | Molecular function | 12 | 169 | 518 | 17548 | 2.4054282515821 | 0.00445018481413415 | 0.227116534924176 | 2.35162195259391 | 0.0231660231660232 | OXSR1//MAP3K9//MAPK4//FGFR3//FGFR2//KIT//RAF1//CDKN1B//SMAD1//GNAZ//IL4R//TIAM1// |
| GO:0001012 | RNA polymerase II regulatory region DNA binding | Molecular function | 32 | 665 | 518 | 17548 | 1.63014486022005 | 0.00474961453334725 | 0.227116534924176 | 2.32334163518212 | 0.0617760617760618 | ARID3B//CUX1//PHOX2A//ASCL1//NFKB1//PURA//REL//CREB3L3//PRDM4//DLX5//ELK1//FOXS1//SATB2//ZNF281//RAX//KCNIP3//SMAD1//MYBL2//NFATC4//PAX5//MED1//CC2D1A//RFX4//SKIL//HLTF//PAX8//FOSL1//PAXBP1//ONECUT2//HEYL//NFYA//BHLHE40// |
| GO:0046873 | metal ion transmembrane transporter activity | Molecular function | 24 | 457 | 518 | 17548 | 1.77906947272374 | 0.00484053853948538 | 0.227116534924176 | 2.31510631775316 | 0.0463320463320463 | KCNJ12//CACNG1//CACNA2D2//SCN3B//SCN1A//SCN7A//KCNA6//KCNQ5//KCNQ4//KCNIP2//KCNIP3//AQP1//ABCC8//SLC6A3//SLC5A3//SLC39A2//SLC30A6//SLC39A8//ATP1B4//SLC5A6//SLC12A7//SLC9A5//GRIN2B//SLC23A2// |
| GO:0000149 | SNARE binding | Molecular function | 10 | 129 | 518 | 17548 | 2.62608123073239 | 0.00493677085311858 | 0.227116534924176 | 2.30655703067711 | 0.0193050193050193 | UNC13B//CPLX1//LRRK2//SNPH//SYT2//SYT9//SYT14//CPLX3//ABCC8//GOSR2// |
| GO:0022838 | substrate-specific channel activity | Molecular function | 23 | 437 | 518 | 17548 | 1.78297094086568 | 0.0055973468399726 | 0.227116534924176 | 2.25201778117274 | 0.0444015444015444 | P2RX2//GRIN2B//AQP1//ANO4//ANO3//BEST1//CHRNA3//HTR3E//CHRFAM7A//KCNJ12//CACNG1//CACNA2D2//SCN3B//SCN1A//SCN7A//KCNA6//KCNQ5//KCNQ4//KCNIP2//SLC26A1//KCNIP3//ABCC8//DLG4// |
| GO:0015267 | channel activity | Molecular function | 24 | 463 | 518 | 17548 | 1.75601457674892 | 0.00567118765884246 | 0.227116534924176 | 2.24632598172981 | 0.0463320463320463 | P2RX2//GRIN2B//AQP1//ANO4//ANO3//BEST1//CHRNA3//HTR3E//CHRFAM7A//KCNJ12//KCNIP3//SCN3B//SCN1A//SCN7A//CACNG1//CACNA2D2//KCNA6//KCNQ5//KCNQ4//KCNIP2//SLC26A1//ABCC8//DLG4//TOMM40L// |
| GO:0022803 | passive transmembrane transporter activity | Molecular function | 24 | 464 | 518 | 17548 | 1.75223006257489 | 0.00582050286459161 | 0.227116534924176 | 2.2350394926898 | 0.0463320463320463 | P2RX2//GRIN2B//AQP1//ANO4//ANO3//BEST1//CHRNA3//HTR3E//CHRFAM7A//KCNJ12//KCNIP3//SCN3B//SCN1A//SCN7A//CACNG1//CACNA2D2//KCNA6//KCNQ5//KCNQ4//KCNIP2//SLC26A1//ABCC8//DLG4//TOMM40L// |
| GO:0005215 | transporter activity | Molecular function | 53 | 1263 | 518 | 17548 | 1.42157698927295 | 0.00602223904469458 | 0.227116534924176 | 2.22024200974466 | 0.102316602316602 | SLC25A2//P2RX2//GRIN2B//AQP1//ANO4//ANO3//BEST1//CHRNA3//HTR3E//CHRFAM7A//KCNJ12//KCNIP3//SCN3B//SCN1A//SCN7A//CACNG1//CACNA2D2//KCNA6//KCNQ5//KCNQ4//KCNIP2//SLC26A1//ABCC8//CPLX1//CPLX3//SLC6A3//SLC5A3//SLC39A2//SLC30A6//SLC39A8//ATP1B4//SLC35A2//SLC25A5//TIMM22//TOMM40L//SLC5A6//PCTP//ATP6V1G2//VPS26A//ASNA1//CTNS//SLC23A2//EBP//RALBP1//SLC27A4//DLG4//SLC10A7//SLC12A7//SLC9A5//TIMM23//SLC52A2//VLDLR//STEAP2// |
| GO:0005244 | voltage-gated ion channel activity | Molecular function | 13 | 198 | 518 | 17548 | 2.22421122421122 | 0.00605642739988205 | 0.227116534924176 | 2.21778348442266 | 0.0250965250965251 | GRIN2B//KCNJ12//CACNG1//CACNA2D2//KCNA6//KCNQ5//KCNQ4//KCNIP2//ABCC8//KCNIP3//SCN3B//SCN1A//SCN7A// |
| GO:0022832 | voltage-gated channel activity | Molecular function | 13 | 198 | 518 | 17548 | 2.22421122421122 | 0.00605642739988205 | 0.227116534924176 | 2.21778348442266 | 0.0250965250965251 | GRIN2B//KCNJ12//KCNIP3//SCN3B//SCN1A//SCN7A//CACNG1//CACNA2D2//KCNA6//KCNQ5//KCNQ4//KCNIP2//ABCC8// |
| GO:0001618 | virus receptor activity | Molecular function | 7 | 74 | 518 | 17548 | 3.2045288531775 | 0.00613354839687205 | 0.227116534924176 | 2.21228820356818 | 0.0135135135135135 | DPP4//CD209//ITGA5//MRC1//NCAM1//SLC52A2//CD81// |
| GO:0104005 | hijacked molecular function | Molecular function | 7 | 74 | 518 | 17548 | 3.2045288531775 | 0.00613354839687205 | 0.227116534924176 | 2.21228820356818 | 0.0135135135135135 | DPP4//CD209//ITGA5//MRC1//NCAM1//SLC52A2//CD81// |
| GO:0015075 | ion transmembrane transporter activity | Molecular function | 40 | 900 | 518 | 17548 | 1.50561990561991 | 0.00659527725893694 | 0.23742998132173 | 2.1807669424231 | 0.0772200772200772 | SLC25A2//P2RX2//GRIN2B//AQP1//ANO4//ANO3//BEST1//CHRNA3//HTR3E//CHRFAM7A//KCNJ12//KCNIP3//SCN3B//SCN1A//SCN7A//CACNG1//CACNA2D2//KCNA6//KCNQ5//KCNQ4//KCNIP2//SLC26A1//ABCC8//SLC6A3//SLC5A3//SLC39A2//SLC30A6//SLC39A8//ATP1B4//SLC25A5//SLC5A6//ATP6V1G2//ASNA1//CTNS//SLC23A2//SLC27A4//DLG4//SLC12A7//SLC9A5//SLC52A2// |
| GO:0000976 | transcription regulatory region sequence-specific DNA binding | Molecular function | 34 | 740 | 518 | 17548 | 1.55648544297193 | 0.00733804895066179 | 0.253017614375921 | 2.13441939547849 | 0.0656370656370656 | ARID3B//CUX1//PHOX2A//ASCL1//NFKB1//PURA//REL//CREB3L3//PRDM4//DLX5//ELK1//FOXS1//SATB2//ZNF281//RAX//KCNIP3//SMAD1//MYBL2//NFATC4//PAX5//MED1//CC2D1A//RFX4//SKIL//HLTF//PAX8//FOSL1//PAXBP1//ONECUT2//HEYL//NFYA//TEAD1//NRIP1//BHLHE40// |
| GO:0017016 | Ras GTPase binding | Molecular function | 26 | 527 | 518 | 17548 | 1.67132380415113 | 0.00763793726049864 | 0.253017614375921 | 2.11702391332734 | 0.0501930501930502 | DLG4//FGFR3//FGFR2//GRIN2B//KIT//NCAM1//SPTBN2//ARHGEF4//BCR//EPS8L2//TIAM1//ARHGEF6//LRRK2//DAPK3//PARD6B//CDC42EP1//UNC13B//MLPH//RALBP1//EXOC4//ARFIP2//ARHGDIB//PAK2//BRK1//WAS//MAPKAP1// |
| GO:0090079 | translation regulator activity, nucleic acid binding | Molecular function | 4 | 27 | 518 | 17548 | 5.01873301873302 | 0.00768242575072945 | 0.253017614375921 | 2.11450162842978 | 0.00772200772200772 | CELF4//PURA//DAZL//LARP1// |
| GO:0000981 | RNA polymerase II transcription factor activity, sequence-specific DNA binding | Molecular function | 47 | 1108 | 518 | 17548 | 1.43699733771936 | 0.00785559754384996 | 0.253017614375921 | 2.10482077432528 | 0.0907335907335907 | TEAD1//FOSL1//HEYL//ARID3B//PRDM4//DLX5//ELK1//SATB2//RAX//SMAD1//MYBL2//PAX5//RFX4//HLTF//PAX8//ONECUT2//FOXS1//ZNF281//KCNIP3//ASCL1//NFATC4//CC2D1A//SKIL//BHLHE40//PAXBP1//NFKB1//REL//HIC1//PURA//TLX2//CREB3L3//FOXP3//FOXG1//OTP//CARHSP1//HOXC6//HOXC9//FOXI2//PHOX2A//NFYA//MED1//MBNL3//CREBZF//HIVEP3//PRDM12//ZNF322//CREB5// |
| GO:0015318 | inorganic molecular entity transmembrane transporter activity | Molecular function | 38 | 856 | 518 | 17548 | 1.503861003861 | 0.00814347632016501 | 0.253017614375921 | 2.08919016214081 | 0.0733590733590734 | SLC25A2//P2RX2//GRIN2B//AQP1//ANO4//ANO3//BEST1//CHRNA3//HTR3E//CHRFAM7A//KCNJ12//CACNG1//CACNA2D2//SCN3B//SCN1A//SCN7A//KCNA6//KCNQ5//KCNQ4//KCNIP2//SLC26A1//KCNIP3//ABCC8//SLC6A3//SLC5A3//SLC39A2//SLC30A6//SLC39A8//ATP1B4//SLC5A6//ATP6V1G2//ASNA1//CTNS//SLC23A2//SLC27A4//DLG4//SLC12A7//SLC9A5// |
| GO:0005178 | integrin binding | Molecular function | 9 | 118 | 518 | 17548 | 2.58379687193247 | 0.00828324161213639 | 0.253017614375921 | 2.08179967061171 | 0.0173745173745174 | GPNMB//EMP2//IL1B//ITGA5//THBS1//TLN1//JAM3//FERMT3//SEMA7A// |
| GO:0051020 | GTPase binding | Molecular function | 31 | 665 | 518 | 17548 | 1.57920283333817 | 0.00839487455105294 | 0.253017614375921 | 2.07598578936423 | 0.0598455598455598 | ARHGEF4//RALGPS2//RALGDS//TIAM1//ARHGEF6//IQSEC3//DLG4//FGFR3//FGFR2//GRIN2B//KIT//NCAM1//SPTBN2//BCR//EPS8L2//MAPKAP1//LRRK2//DAPK3//PARD6B//CDC42EP1//UNC13B//MLPH//RALBP1//EXOC4//PAK2//PEX5//WAS//ARFIP2//ARHGDIB//BRK1//SH3GL1// |
| GO:0016411 | acylglycerol O-acyltransferase activity | Molecular function | 4 | 29 | 518 | 17548 | 4.67261350019971 | 0.009926572811753 | 0.292382690091634 | 2.00320066750201 | 0.00772200772200772 | AGPAT1//LCLAT1//ABHD5//MOGAT2// |
| GO:0015079 | potassium ion transmembrane transporter activity | Molecular function | 11 | 166 | 518 | 17548 | 2.24482485928269 | 0.0103929051928249 | 0.299315669553357 | 1.98326303445922 | 0.0212355212355212 | KCNJ12//KCNA6//KCNQ5//KCNQ4//KCNIP2//KCNIP3//AQP1//ABCC8//ATP1B4//SLC12A7//SLC9A5// |
| GO:0031267 | small GTPase binding | Molecular function | 27 | 570 | 518 | 17548 | 1.60467384677911 | 0.0110163179925621 | 0.306588293720686 | 1.95796353640202 | 0.0521235521235521 | DLG4//FGFR3//FGFR2//GRIN2B//KIT//NCAM1//SPTBN2//ARHGEF4//BCR//EPS8L2//TIAM1//ARHGEF6//MAPKAP1//LRRK2//DAPK3//PARD6B//CDC42EP1//UNC13B//MLPH//RALBP1//EXOC4//ARFIP2//ARHGDIB//PAK2//BRK1//WAS//PEX5// |
| GO:0042802 | identical protein binding | Molecular function | 65 | 1661 | 518 | 17548 | 1.32568880913252 | 0.0113031963146651 | 0.306588293720686 | 1.94679872947819 | 0.125482625482625 | TIRAP//LRRK2//MYOM3//DAPK3//EXD1//DPP4//FGFR2//HEYL//HMOX1//IMPA2//KIT//CCDC103//SMAD1//ASCL1//MAP3K9//NDP//NFKB1//FOXP3//PML//MAPK4//PRTFDC1//RAG1//TTK//JAM3//CREB3L3//TRIM5//BHLHE40//NFS1//CD79A//PTPRU//SYT9//CTH//DAZL//DLAT//ALAD//EWSR1//FGFR3//MAPRE1//P2RX2//ESYT1//ARFIP2//SULT4A1//PACSIN1//MCIDAS//AQP1//PAK2//SH3GLB2//CREBZF//RAF1//SFRP1//SH3GL1//SNRPA//TRIM21//CNTN2//THBS1//BEST1//WAS//YWHAB//DDX39B//PIP4K2C//RNF135//DDI2//CD74//SEPT7//OXSR1// |
| GO:1901981 | phosphatidylinositol phosphate binding | Molecular function | 10 | 147 | 518 | 17548 | 2.30452026370394 | 0.0119707166955866 | 0.306588293720686 | 1.9218798472861 | 0.0193050193050193 | TIRAP//SYT9//MARK1//MAPKAP1//SESTD1//FERMT2//NRGN//OGT//SNX19//ARFIP2// |
| GO:0022890 | inorganic cation transmembrane transporter activity | Molecular function | 28 | 601 | 518 | 17548 | 1.57827045015065 | 0.0119757853212958 | 0.306588293720686 | 1.92169599778627 | 0.0540540540540541 | P2RX2//GRIN2B//AQP1//KCNJ12//CACNG1//CACNA2D2//SCN3B//SCN1A//SCN7A//KCNA6//KCNQ5//KCNQ4//KCNIP2//KCNIP3//ABCC8//SLC6A3//SLC5A3//SLC39A2//SLC30A6//SLC39A8//ATP1B4//SLC5A6//ATP6V1G2//SLC12A7//SLC9A5//CHRNA3//HTR3E//SLC23A2// |
| GO:0017147 | Wnt-protein binding | Molecular function | 4 | 31 | 518 | 17548 | 4.37115456470295 | 0.0125570197066971 | 0.306588293720686 | 1.9011134241754 | 0.00772200772200772 | FZD2//TRABD2B//SFRP1//FZD3// |
| GO:0008320 | protein transmembrane transporter activity | Molecular function | 3 | 17 | 518 | 17548 | 5.97819668407904 | 0.0127745122383619 | 0.306588293720686 | 1.89365567328577 | 0.00579150579150579 | TIMM23//TIMM22//TOMM40L// |
| GO:0008391 | arachidonic acid monooxygenase activity | Molecular function | 3 | 17 | 518 | 17548 | 5.97819668407904 | 0.0127745122383619 | 0.306588293720686 | 1.89365567328577 | 0.00579150579150579 | CYP2C18//CYP2S1//CYP4F2// |
| GO:0008392 | arachidonic acid epoxygenase activity | Molecular function | 3 | 17 | 518 | 17548 | 5.97819668407904 | 0.0127745122383619 | 0.306588293720686 | 1.89365567328577 | 0.00579150579150579 | CYP2C18//CYP2S1//CYP4F2// |
| GO:0030676 | Rac guanyl-nucleotide exchange factor activity | Molecular function | 3 | 17 | 518 | 17548 | 5.97819668407904 | 0.0127745122383619 | 0.306588293720686 | 1.89365567328577 | 0.00579150579150579 | ARHGEF4//EPS8L2//TIAM1// |
| GO:0005261 | cation channel activity | Molecular function | 17 | 316 | 518 | 17548 | 1.82246713259371 | 0.0130341998228855 | 0.307133144917447 | 1.88491562526835 | 0.0328185328185328 | P2RX2//GRIN2B//AQP1//KCNJ12//CACNG1//CACNA2D2//SCN3B//SCN1A//SCN7A//KCNA6//KCNQ5//KCNQ4//KCNIP2//KCNIP3//ABCC8//CHRNA3//HTR3E// |
| GO:0016740 | transferase activity | Molecular function | 90 | 2437 | 518 | 17548 | 1.25107932247858 | 0.0135128136605968 | 0.31272511614524 | 1.86925421204064 | 0.173745173745174 | POLR2E//ST6GALNAC6//GAL3ST1//TGM4//TGM2//ST8SIA5//ST3GAL3//AGPAT1//LCLAT1//ABHD5//MOGAT2//POLR2M//PARP16//TNKS2//DGKG//CTH//SULT4A1//DLG4//PRTFDC1//GALNT3//GALNT10//PGGT1B//LRRK2//PAK2//PRKAA1//PRKAB1//RAF1//SGK2//NEK7//DAPK3//MARK1//MAP3K9//PIM1//MAPK4//BCR//TTK//AATK//OXSR1//CAMKK2//CDKN1B//FGFR3//FGFR2//KIT//CUX1//DLAT//MAEA//RNF38//RNF217//FBXO45//MGRN1//FBXL3//FBXO10//RLIM//TRIM68//RNF114//KLHL14//RAG1//RNF123//SKP1//TRIM21//UBE2D3//UBE2E1//RNF135//KLHL22//FBXL20//TRIM5//UCK2//UCK1//EFNA3//NAT9//HS6ST1//PRDM4//PRDM12//FTSJ1//OGT//GLYCTK//PIP4K2C//URM1//KMT2E//GOLGA7B//MGAT5//NFS1//UBE4B//FAM57B//RNF149//MED1//RNF125//UBE2Z//HLTF//TRIM7// |
| GO:1990837 | sequence-specific double-stranded DNA binding | Molecular function | 34 | 776 | 518 | 17548 | 1.48427735541138 | 0.0143030942893501 | 0.325207196473644 | 1.84456999837975 | 0.0656370656370656 | NFKB1//CREB3L3//ARID3B//CUX1//PHOX2A//ASCL1//PURA//REL//PRDM4//DLX5//ELK1//FOXS1//SATB2//ZNF281//RAX//KCNIP3//SMAD1//MYBL2//NFATC4//PAX5//MED1//CC2D1A//RFX4//SKIL//HLTF//PAX8//FOSL1//PAXBP1//ONECUT2//HEYL//NFYA//TEAD1//NRIP1//BHLHE40// |
| GO:0061630 | ubiquitin protein ligase activity | Molecular function | 13 | 224 | 518 | 17548 | 1.96604384997242 | 0.015947888773636 | 0.356352825010901 | 1.79729680192641 | 0.0250965250965251 | UBE4B//RNF38//RNF217//MGRN1//RNF149//RLIM//MED1//RNF125//RNF114//RAG1//SKP1//UBE2Z//HLTF// |
| GO:0003841 | 1-acylglycerol-3-phosphate O-acyltransferase activity | Molecular function | 3 | 19 | 518 | 17548 | 5.34891282259703 | 0.0174281435664933 | 0.358521810510719 | 1.75874887116691 | 0.00579150579150579 | AGPAT1//LCLAT1//ABHD5// |
| GO:0005229 | intracellular calcium activated chloride channel activity | Molecular function | 3 | 19 | 518 | 17548 | 5.34891282259703 | 0.0174281435664933 | 0.358521810510719 | 1.75874887116691 | 0.00579150579150579 | ANO4//ANO3//BEST1// |
| GO:0022884 | macromolecule transmembrane transporter activity | Molecular function | 3 | 19 | 518 | 17548 | 5.34891282259703 | 0.0174281435664933 | 0.358521810510719 | 1.75874887116691 | 0.00579150579150579 | TIMM22//TOMM40L//TIMM23// |
| GO:0038191 | neuropilin binding | Molecular function | 3 | 19 | 518 | 17548 | 5.34891282259703 | 0.0174281435664933 | 0.358521810510719 | 1.75874887116691 | 0.00579150579150579 | SEMA5B//SEMA4G//SEMA7A// |
| GO:0061778 | intracellular chloride channel activity | Molecular function | 3 | 19 | 518 | 17548 | 5.34891282259703 | 0.0174281435664933 | 0.358521810510719 | 1.75874887116691 | 0.00579150579150579 | ANO4//ANO3//BEST1// |
| GO:0008289 | lipid binding | Molecular function | 31 | 707 | 518 | 17548 | 1.48538880363491 | 0.0186223917034739 | 0.368464884761995 | 1.72996454263948 | 0.0598455598455598 | PTAFR//SYT9//MARK1//THBS1//TLN1//UNC13B//ESYT1//ARFIP2//PACSIN1//PAFAH2//BIN2//SPTBN2//PLA2G4C//SYT2//SYT14//WDFY1//TIRAP//MAPKAP1//SESTD1//FERMT2//NRGN//OGT//ALDH1A2//PGGT1B//PCTP//SNX19//TULP4//DBI//SH3GL1//TIAM1//ACBD6// |
| GO:0061659 | ubiquitin-like protein ligase activity | Molecular function | 13 | 229 | 518 | 17548 | 1.9231171283573 | 0.018789796455709 | 0.368464884761995 | 1.72607792445728 | 0.0250965250965251 | UBE4B//RNF38//RNF217//MGRN1//RNF149//RLIM//MED1//RNF125//RNF114//RAG1//SKP1//UBE2Z//HLTF// |
| GO:0001077 | transcriptional activator activity, RNA polymerase II proximal promoter sequence-specific DNA binding | Molecular function | 15 | 279 | 518 | 17548 | 1.82131440195956 | 0.0190434906696971 | 0.368464884761995 | 1.72025344252509 | 0.028957528957529 | ARID3B//PRDM4//DLX5//ELK1//SATB2//HEYL//RAX//SMAD1//MYBL2//PAX5//RFX4//HLTF//PAX8//FOSL1//ONECUT2// |
| GO:0043565 | sequence-specific DNA binding | Molecular function | 46 | 1139 | 518 | 17548 | 1.36814451476436 | 0.0190487247523562 | 0.368464884761995 | 1.7201340935607 | 0.0888030888030888 | NFKB1//CREB3L3//ARID3B//CUX1//PHOX2A//ASCL1//PURA//REL//PRDM4//DLX5//ELK1//FOXS1//SATB2//ZNF281//RAX//KCNIP3//SMAD1//MYBL2//NFATC4//PAX5//MED1//CC2D1A//RFX4//SKIL//HLTF//PAX8//FOSL1//PAXBP1//ONECUT2//HEYL//NFYA//TEAD1//NRIP1//BHLHE40//FOXG1//SIRT3//OTP//HIC1//TLX2//HOXC6//HOXC9//FOXI2//FOXP3//CALCOCO1//RAG1//HIVEP3// |
| GO:0042171 | lysophosphatidic acid acyltransferase activity | Molecular function | 3 | 20 | 518 | 17548 | 5.08146718146718 | 0.0200632447844802 | 0.376840075951976 | 1.69759882814333 | 0.00579150579150579 | AGPAT1//LCLAT1//ABHD5// |
| GO:0071617 | lysophospholipid acyltransferase activity | Molecular function | 3 | 20 | 518 | 17548 | 5.08146718146718 | 0.0200632447844802 | 0.376840075951976 | 1.69759882814333 | 0.00579150579150579 | AGPAT1//LCLAT1//ABHD5// |
| GO:0001614 | purinergic nucleotide receptor activity | Molecular function | 3 | 21 | 518 | 17548 | 4.83949255377827 | 0.022905109041137 | 0.39762504509526 | 1.64006763639821 | 0.00579150579150579 | GPR34//PTAFR//P2RX2// |
| GO:0008373 | sialyltransferase activity | Molecular function | 3 | 21 | 518 | 17548 | 4.83949255377827 | 0.022905109041137 | 0.39762504509526 | 1.64006763639821 | 0.00579150579150579 | ST6GALNAC6//ST8SIA5//ST3GAL3// |
| GO:0016502 | nucleotide receptor activity | Molecular function | 3 | 21 | 518 | 17548 | 4.83949255377827 | 0.022905109041137 | 0.39762504509526 | 1.64006763639821 | 0.00579150579150579 | P2RX2//GPR34//PTAFR// |
| GO:0005547 | phosphatidylinositol-3,4,5-trisphosphate binding | Molecular function | 4 | 37 | 518 | 17548 | 3.66231868934572 | 0.0229614383353426 | 0.39762504509526 | 1.6390009106322 | 0.00772200772200772 | FERMT2//NRGN//MAPKAP1//OGT// |
| GO:0017080 | sodium channel regulator activity | Molecular function | 4 | 37 | 518 | 17548 | 3.66231868934572 | 0.0229614383353426 | 0.39762504509526 | 1.6390009106322 | 0.00772200772200772 | RSC1A1//SGK2//SCN3B//PRSS8// |
| GO:0008324 | cation transmembrane transporter activity | Molecular function | 29 | 663 | 518 | 17548 | 1.48177524648113 | 0.0230107086281979 | 0.39762504509526 | 1.63807000677346 | 0.055984555984556 | SLC25A2//P2RX2//GRIN2B//AQP1//KCNJ12//CACNG1//CACNA2D2//SCN3B//SCN1A//SCN7A//KCNA6//KCNQ5//KCNQ4//KCNIP2//KCNIP3//ABCC8//SLC6A3//SLC5A3//SLC39A2//SLC30A6//SLC39A8//ATP1B4//SLC5A6//ATP6V1G2//SLC12A7//SLC9A5//CHRNA3//HTR3E//SLC23A2// |
| GO:0035091 | phosphatidylinositol binding | Molecular function | 13 | 237 | 518 | 17548 | 1.85820178225242 | 0.0241087070657097 | 0.405801055074685 | 1.61782607992882 | 0.0250965250965251 | WDFY1//TIRAP//SYT9//MARK1//MAPKAP1//SESTD1//FERMT2//NRGN//OGT//SNX19//ARFIP2//TULP4//TLN1// |
| GO:0001078 | transcriptional repressor activity, RNA polymerase II proximal promoter sequence-specific DNA binding | Molecular function | 9 | 141 | 518 | 17548 | 2.1623264601988 | 0.024110093549962 | 0.405801055074685 | 1.61780110450627 | 0.0173745173745174 | FOXS1//ZNF281//KCNIP3//ASCL1//NFATC4//CC2D1A//SKIL//BHLHE40//PAXBP1// |
| GO:0015077 | monovalent inorganic cation transmembrane transporter activity | Molecular function | 19 | 393 | 518 | 17548 | 1.63779264542623 | 0.0248313764701597 | 0.412582870581115 | 1.60499920566388 | 0.0366795366795367 | KCNJ12//SCN3B//SCN1A//SCN7A//KCNA6//KCNQ5//KCNQ4//KCNIP2//KCNIP3//AQP1//ABCC8//SLC6A3//SLC5A3//ATP1B4//SLC5A6//ATP6V1G2//SLC12A7//SLC9A5//SLC23A2// |
| GO:0005385 | zinc ion transmembrane transporter activity | Molecular function | 3 | 22 | 518 | 17548 | 4.61951561951562 | 0.0259538239243973 | 0.412699361577548 | 1.58579864602677 | 0.00579150579150579 | SLC39A2//SLC30A6//SLC39A8// |
| GO:0015464 | acetylcholine receptor activity | Molecular function | 3 | 22 | 518 | 17548 | 4.61951561951562 | 0.0259538239243973 | 0.412699361577548 | 1.58579864602677 | 0.00579150579150579 | CHRM1//CHRNA3//CHRFAM7A// |
| GO:0042813 | Wnt-activated receptor activity | Molecular function | 3 | 22 | 518 | 17548 | 4.61951561951562 | 0.0259538239243973 | 0.412699361577548 | 1.58579864602677 | 0.00579150579150579 | FZD2//SFRP1//FZD3// |
| GO:0022843 | voltage-gated cation channel activity | Molecular function | 9 | 143 | 518 | 17548 | 2.13208413208413 | 0.0261121509640115 | 0.412699361577548 | 1.5831573521018 | 0.0173745173745174 | GRIN2B//KCNJ12//CACNG1//CACNA2D2//KCNA6//KCNQ5//KCNQ4//KCNIP2//ABCC8// |
| GO:0015238 | drug transmembrane transporter activity | Molecular function | 7 | 99 | 518 | 17548 | 2.3953043953044 | 0.0272362106897044 | 0.421456830606144 | 1.56485331493201 | 0.0135135135135135 | SLC25A2//SLC6A3//SLC25A5//AQP1//SLC52A2//EBP//RALBP1// |
| GO:0017069 | snRNA binding | Molecular function | 4 | 39 | 518 | 17548 | 3.47450747450747 | 0.027316646428176 | 0.421456830606144 | 1.56357261857525 | 0.00772200772200772 | DDX39B//SNRPA//SNRPB//LSM11// |
| GO:0005248 | voltage-gated sodium channel activity | Molecular function | 3 | 23 | 518 | 17548 | 4.41866711431929 | 0.0292088654493918 | 0.431972655895323 | 1.53448531186034 | 0.00579150579150579 | SCN3B//SCN1A//SCN7A// |
| GO:0017134 | fibroblast growth factor binding | Molecular function | 3 | 23 | 518 | 17548 | 4.41866711431929 | 0.0292088654493918 | 0.431972655895323 | 1.53448531186034 | 0.00579150579150579 | FGFR3//FGFR2//THBS1// |
| GO:0015276 | ligand-gated ion channel activity | Molecular function | 9 | 146 | 518 | 17548 | 2.0882741841646 | 0.0293314766348676 | 0.431972655895323 | 1.53266607277696 | 0.0173745173745174 | P2RX2//GRIN2B//AQP1//CHRNA3//HTR3E//CHRFAM7A//KCNJ12//ABCC8//DLG4// |
| GO:0022834 | ligand-gated channel activity | Molecular function | 9 | 146 | 518 | 17548 | 2.0882741841646 | 0.0293314766348676 | 0.431972655895323 | 1.53266607277696 | 0.0173745173745174 | P2RX2//GRIN2B//AQP1//CHRNA3//HTR3E//CHRFAM7A//KCNJ12//ABCC8//DLG4// |
| GO:0005072 | transforming growth factor beta receptor, cytoplasmic mediator activity | Molecular function | 2 | 10 | 518 | 17548 | 6.77528957528957 | 0.033451169676801 | 0.481696843345934 | 1.47558869178857 | 0.00386100386100386 | SMAD1//CDKN1B// |
| GO:0017128 | phospholipid scramblase activity | Molecular function | 2 | 10 | 518 | 17548 | 6.77528957528957 | 0.033451169676801 | 0.481696843345934 | 1.47558869178857 | 0.00386100386100386 | ANO4//ANO3// |
| GO:0003690 | double-stranded DNA binding | Molecular function | 35 | 860 | 518 | 17548 | 1.37869264613451 | 0.0343794673714612 | 0.489023641677062 | 1.46370085596273 | 0.0675675675675676 | NFKB1//CREB3L3//ARID3B//CUX1//PHOX2A//ASCL1//PURA//REL//PRDM4//DLX5//ELK1//FOXS1//SATB2//ZNF281//RAX//KCNIP3//SMAD1//MYBL2//NFATC4//PAX5//MED1//CC2D1A//RFX4//SKIL//HLTF//PAX8//FOSL1//PAXBP1//ONECUT2//HEYL//NFYA//TEAD1//NRIP1//BHLHE40//HMGB3// |
| GO:0001786 | phosphatidylserine binding | Molecular function | 4 | 42 | 518 | 17548 | 3.22632836918551 | 0.0347146412301618 | 0.489023641677062 | 1.45948731874291 | 0.00772200772200772 | SYT9//MARK1//THBS1//TLN1// |
| GO:0035586 | purinergic receptor activity | Molecular function | 3 | 25 | 518 | 17548 | 4.06517374517375 | 0.036333061981553 | 0.500932429022263 | 1.43969800027503 | 0.00579150579150579 | P2RX2//GPR34//PTAFR// |
| GO:0090482 | vitamin transmembrane transporter activity | Molecular function | 3 | 25 | 518 | 17548 | 4.06517374517375 | 0.036333061981553 | 0.500932429022263 | 1.43969800027503 | 0.00579150579150579 | SLC5A6//SLC23A2//SLC52A2// |
| GO:0005267 | potassium channel activity | Molecular function | 8 | 129 | 518 | 17548 | 2.10086498458592 | 0.0375038693962057 | 0.511631734078764 | 1.42592392238619 | 0.0154440154440154 | KCNJ12//KCNA6//KCNQ5//KCNQ4//KCNIP2//ABCC8//KCNIP3//AQP1// |
| GO:0005200 | structural constituent of cytoskeleton | Molecular function | 7 | 107 | 518 | 17548 | 2.21621621621622 | 0.0391712326127361 | 0.521388175551495 | 1.40703276216449 | 0.0135135135135135 | ACTR3//ACTR2//KRT2//KRT9//SPTBN2//TLN1//CCDC6// |
| GO:0015924 | mannosyl-oligosaccharide mannosidase activity | Molecular function | 2 | 11 | 518 | 17548 | 6.15935415935416 | 0.0400991771637097 | 0.521388175551495 | 1.39686453902388 | 0.00386100386100386 | MAN1C1//EDEM3// |
| GO:0033130 | acetylcholine receptor binding | Molecular function | 2 | 11 | 518 | 17548 | 6.15935415935416 | 0.0400991771637097 | 0.521388175551495 | 1.39686453902388 | 0.00386100386100386 | UBXN2A//DLG4// |
| GO:0072509 | divalent inorganic cation transmembrane transporter activity | Molecular function | 3 | 26 | 518 | 17548 | 3.90882090882091 | 0.0401985329686518 | 0.521388175551495 | 1.39578979605098 | 0.00579150579150579 | SLC39A2//SLC30A6//SLC39A8// |
| GO:0016709 | oxidoreductase activity, acting on paired donors, with incorporation or reduction of molecular oxygen, NAD(P)H as one donor, and incorporation of one atom of oxygen | Molecular function | 4 | 44 | 518 | 17548 | 3.07967707967708 | 0.0402305691011956 | 0.521388175551495 | 1.39544382386024 | 0.00772200772200772 | FAXDC2//CYP4F3//CYP4F2//MICAL2// |
| GO:0140096 | catalytic activity, acting on a protein | Molecular function | 84 | 2370 | 518 | 17548 | 1.20068422853233 | 0.0410886847965161 | 0.527160322588218 | 1.38627775980304 | 0.162162162162162 | PPIH//TGM4//TGM2//HTRA4//PRSS8//DDI2//USP37//SFRP1//CASP7//MMP24//COPS5//TRABD2B//DPP4//TMPRSS2//C1QA//GALNT3//GALNT10//PGGT1B//LRRK2//PAK2//PRKAA1//PRKAB1//RAF1//SGK2//NEK7//DAPK3//MARK1//MAP3K9//PIM1//MAPK4//BCR//TTK//AATK//OXSR1//CAMKK2//CDKN1B//FGFR3//FGFR2//KIT//CUX1//TAB1//PPP3CB//PTPRU//PTPRT//PTPN18//DLAT//TTL//MAEA//RNF38//RNF217//FBXO45//MGRN1//FBXL3//FBXO10//RLIM//TRIM68//RNF114//KLHL14//RAG1//RNF123//SKP1//TRIM21//UBE2D3//UBE2E1//RNF135//KLHL22//FBXL20//TRIM5//UCHL5//USP46//USP45//EFNA3//OGT//SIRT3//KMT2E//GOLGA7B//MGAT5//UBE4B//USP54//RNF149//MED1//RNF125//UBE2Z//HLTF// |
| GO:0001228 | transcriptional activator activity, RNA polymerase II transcription regulatory region sequence-specific DNA binding | Molecular function | 19 | 417 | 518 | 17548 | 1.54353119820746 | 0.041489469833332 | 0.527160322588218 | 1.38206211469933 | 0.0366795366795367 | ARID3B//PRDM4//DLX5//ELK1//SATB2//HEYL//RAX//SMAD1//MYBL2//PAX5//RFX4//HLTF//PAX8//FOSL1//ONECUT2//NFKB1//REL//TLX2//CREB3L3// |
| GO:0099094 | ligand-gated cation channel activity | Molecular function | 7 | 109 | 518 | 17548 | 2.17555169848748 | 0.042614892264279 | 0.536202916257336 | 1.37043860517019 | 0.0135135135135135 | P2RX2//GRIN2B//AQP1//KCNJ12//ABCC8//CHRNA3//HTR3E// |
| GO:0030165 | PDZ domain binding | Molecular function | 6 | 87 | 518 | 17548 | 2.33630675009985 | 0.0437172865858528 | 0.541922465021542 | 1.35934680137378 | 0.0115830115830116 | DLG4//FZD2//CCDC88C//EXOC4//SHISA9//FZD3// |
| GO:0061631 | ubiquitin conjugating enzyme activity | Molecular function | 3 | 27 | 518 | 17548 | 3.76404976404976 | 0.0442630463708921 | 0.541922465021542 | 1.3539586993889 | 0.00579150579150579 | UBE2Z//UBE2D3//UBE2E1// |
| GO:0001227 | transcriptional repressor activity, RNA polymerase II transcription regulatory region sequence-specific DNA binding | Molecular function | 12 | 233 | 518 | 17548 | 1.74470976187714 | 0.0443239053181199 | 0.541922465021542 | 1.35336198150642 | 0.0231660231660232 | FOXS1//ZNF281//KCNIP3//ASCL1//NFATC4//CC2D1A//SKIL//BHLHE40//PAXBP1//HIC1//NFKB1//PURA// |
| GO:0004861 | cyclin-dependent protein serine/threonine kinase inhibitor activity | Molecular function | 2 | 12 | 518 | 17548 | 5.64607464607465 | 0.0471968169526214 | 0.561165823583462 | 1.3260872900635 | 0.00386100386100386 | CDKN1B//CDKN2A// |
| GO:0016783 | sulfurtransferase activity | Molecular function | 2 | 12 | 518 | 17548 | 5.64607464607465 | 0.0471968169526214 | 0.561165823583462 | 1.3260872900635 | 0.00386100386100386 | NFS1//URM1// |
| GO:0042975 | peroxisome proliferator activated receptor binding | Molecular function | 2 | 12 | 518 | 17548 | 5.64607464607465 | 0.0471968169526214 | 0.561165823583462 | 1.3260872900635 | 0.00386100386100386 | NFATC4//MED1// |
| GO:0005546 | phosphatidylinositol-4,5-bisphosphate binding | Molecular function | 5 | 67 | 518 | 17548 | 2.52809312510805 | 0.0476642399076237 | 0.561488457163133 | 1.32180732819636 | 0.00965250965250965 | TIRAP//SYT9//MARK1//MAPKAP1//SESTD1// |
| GO:0061650 | ubiquitin-like protein conjugating enzyme activity | Molecular function | 3 | 28 | 518 | 17548 | 3.6296194153337 | 0.0485236938289127 | 0.561488457163133 | 1.31404614621328 | 0.00579150579150579 | UBE2Z//UBE2D3//UBE2E1// |
| GO:1904680 | peptide transmembrane transporter activity | Molecular function | 3 | 28 | 518 | 17548 | 3.6296194153337 | 0.0485236938289127 | 0.561488457163133 | 1.31404614621328 | 0.00579150579150579 | TIMM22//TOMM40L//TIMM23// |
| GO:0015081 | sodium ion transmembrane transporter activity | Molecular function | 9 | 161 | 518 | 17548 | 1.89371447756541 | 0.049619832855008 | 0.569091180354782 | 1.30434470298743 | 0.0173745173745174 | SCN3B//SCN1A//SCN7A//SLC6A3//SLC5A3//ATP1B4//SLC5A6//SLC9A5//SLC23A2// |
